# Perceived Insider Status and Feedback Reactions: A Dual Path of Feedback Motivation Attribution

**DOI:** 10.3389/fpsyg.2017.00668

**Published:** 2017-05-01

**Authors:** Xiao Chen, JianQiao Liao, Weijiong Wu, Wei Zhang

**Affiliations:** ^1^School of Management, Huazhong University of Science and TechnologyWuhan, China; ^2^School of Management, Zhejiang University of Finance and EconomicsHangzhou, China; ^3^School of Hydropower and Information Engineering, Huazhong University of Science and TechnologyWuhan, China

**Keywords:** perceived insider status (PIS), feedback reactions, feedback motivation attribution

## Abstract

Many studies have evaluated how the characteristics of feedback receiver, feedback deliverer and feedback information influence psychological feedback reactions of the feedback receiver while largely neglecting that feedback intervention is a kind of social interaction process. To address this issue, this study proposes that employees’ perceived insider status (PIS), as a kind of employee-organization relationship, could also influence employees’ reactions to supervisory feedback. In particular, this study investigates the influence of PIS focusing on affective and cognitive feedback reactions, namely feedback satisfaction and feedback utility. Surveys were conducted in a machinery manufacturing company in the Guangdong province of China. Samples were collected from 192 employees. Data analysis demonstrated that PIS and feedback utility possessed a U-shaped relationship, whereas PIS and feedback satisfaction exhibited positively linear relationships. The analysis identified two kinds of mediating mechanisms related to feedback satisfaction and feedback utility. Internal feedback motivation attribution partially mediated the relationship between PIS and feedback satisfaction but failed to do the same with respect to the relationship between PIS and feedback utility. In contrast, external feedback motivation attribution partially mediated the relationship between PIS and feedback utility while failing to mediate the relationship between PIS and feedback satisfaction. Theoretical contributions and practical implications of the findings are discussed at the end of the paper.

## Introduction

Performance feedback has been widely acknowledged as a vital organizational practice that is capable of improving employee performance and behavior. Despite the broad use of performance feedback across organizations, the effectiveness of feedback intervention (supervisors use feedback to modify employees’ work performance and behaviors) has not yet been assessed adequately. A highly cited meta analysis (Kluger and DeNisi, 1996) reported that approximately one third of feedback intervention had resulted in decreased worker performance. To understand why the effectiveness of feedback intervention is unstable, it is necessary to understand the recipients’ psychological reactions to feedback. For this purpose, scholars conceptualized the constructs of feedback reactions and attempted to measure them by utilizing the dimensions of cognition and emotion (Audia and Locke, 2003; Jawahar, 2006). Several researchers have studied the antecedents of feedback reactions from three perspectives: individual differences of feedback recipients, properties of feedback information, and characteristics of feedback deliverers (Ilgen et al., 1979). By reviewing available researches, supervisors might understand that the effectiveness of that feedback could be improved by enhancing the quality of feedback information (Whitaker and Levy, 2012), delivering customized feedback to specific individuals (Mulder et al., 2013) or altering certain characteristics such as increasing their own power or reliability (Fedor et al., 2001; Tuytens and Devos, 2012).

Several investigators have separately examined the properties of different components (feedback information, feedback deliverer, feedback recipient) during feedback communication as antecedents of feedback reactions, but neglected that feedback intervention is essentially a form of social interaction (Hempel, 2008; Pichler, 2012). Being the basis of social interaction, social relationships could also influence feedback reactions. It has been demonstrated that one reason supervisors find it difficult to provide feedback is because they are unable to overlook the critical influence of social relationships (Hempel, 2008). Some studies incorporated interactions between supervisors and subordinates and demonstrated that predefined dyadic level interpersonal relationships influence feedback reactions. In addition to this, employees may develop perceptions about social relationships by contacting other group members. These processes are often referred to as group dynamics (Levy and Williams, 2004). Through in-group dynamic social interaction, employees can develop knowledge about self-organization relationships—perceived insider status, PIS (Stamper and Masterson, 2002). How this kind of knowledge might influence employees’ feedback reactions to supervisory feedback is the research question addressed in the present study.

Perception of self-organization relationship is one kind of ‘self-process,’ the “self-process” could also be related to feedback reactions, e.g., self-esteem defined as evaluation of oneself has been proposed to be one of the antecedents of feedback reactions (McFarlin and Blascovich, 1981). Although self-esteem looks at self-processes from a static, trait-like perspective, another way of evaluating how a self-process would influence feedback process is to consider the interaction between self-identity and social context (self-organization relationship). Individuals could enable the formation of self-organization relationships through a self-categorization process (Turner et al., 1987). According to the theory of self-categorization (Turner et al., 1987), individuals would develop certain bonds between self-identity and outside world (e.g., group, organization), through which individuals could establish different strengths of self-organization relationships. These varieties of self-organization relationships could lead to a range of reactions from individuals concerning information and people in the group.

According to self-categorization theory, employees develop a PIS through conceptions about their social identities in relation to coworkers (Stamper and Masterson, 2002). Perceived group memberships play a role in the formation of self-identity, which automatically exerts influence on cognition and behavior. For instance, individuals possessing a group identity would perceive themselves as enjoying more benefits than relative outsiders (Turner et al., 1987). When a member feels that he/she has been treated as an insider (PIS), he or she will treat supervisory feedback as high quality information or a resource for future performance development. By contrast, those perceiving themselves as enjoying a relative low insider status are likely to have different opinions and treat supervisory feedback as unimportant or useless message. Notwithstanding the existence of a variety of perceptions about supervisory feedback, employees can still generate different judgments about supervisors. As described by self-categorization theory, people tend to make non-constructive intention attribution with respect to feedback providers who are perceived as outsiders (Hornsey et al., 2005). When an employee perceives a low insider status (perceive oneself as an outsider), the supervisors might be viewed as relative outsiders, thus leading him/her to negatively attribute supervisors’ feedback motivation and generate negative feedback reactions.

In view of the above arguments, we use self-categorization as our overarching theory. We assume that, through self-categorization process, the variously developed self-organization relationships (PIS) would influence employee reactions to supervisory feedback and judgments about their supervisors. For the purpose of this study, PIS was selected as the independent variable. We believe that PIS is a type of information derived from social interactions and represent the self-organization relationship. We also consider that attribution about supervisory feedback motivation could be the mechanism linking PIS and feedback reactions. The hope is that the discovery of the relationship between PIS and feedback reactions would lead to contributions revealing the impact of social elements on feedback reactions.

## Development of Hypotheses

### Feedback Reactions

Reactions to supervisory feedback may be classified along two dimensions: affective and cognitive reactions. Affective feedback reactions refer to how the feedback makes an employee feel. It is measured by either asking about the intensity of generated emotions (Belschak and Den Hartog, 2009) or about emotional attitudes such as satisfaction with the appraisal system in use (Dobbins et al., 1990). Cognitive reactions refer broadly to what an employee thinks about a given feedback event. In many situations, cognitive reaction is measured by assessing to what degree workers view the feedback as being useful to their own development (commonly referred to as feedback utility) (Jawahar, 2010).

Building on the theoretical model proposed by Ilgen (Ilgen et al., 1979), effectiveness of feedback intervention is determined by feedback recipients’ perceptions concerning feedback utility. Only when the employee has determined that the supervisory feedback is valuable will he/she use the information for actual performance improvement. However, according to feedback intervention theory (Kluger and DeNisi, 1996), when employees receive feedback that might threaten their self-esteem, negative emotional reactions may be triggered, which may adversely affect future performance (Anseel et al., 2011). These two theories imply that affective and cognitive feedback reactions are of similar importance with regard to the effectiveness of feedback intervention. Many scholars have studied these two aspects either separately or combined them together into a single construct (Culbertson et al., 2013; Wang et al., 2015). However, according to the cognitive theory of emotion (Oatley and Johnson-laird, 1987), behavior is generally influenced through interactions of emotion and cognition. In certain circumstances, employees may not be happy with the feedback. However, as long as they perceive it as being valuable, they will overlook their negative feelings and respond to feedback by striving to improve their job performance. Therefore, we treat affective feedback reactions and cognitive reactions as different dimensions and utilize distinct correlation patterns to explain employee psychological feedback processing.

Some previous investigators had focused on the attributes of feedback, such as the quality of feedback information, the manner of feedback delivery, and the attributes of the feedback deliverer (Steelman et al., 2004; Tuytens and Devos, 2012). The theoretical foundation of many of these studies consisted of the model proposed by Ilgen, which treats feedback intervention as different components of information communication (Ilgen et al., 1979). However, all types of information communication are grounded essentially in social interactions. As the bases of social interaction, social relationships could influence feedback reactions. In this study, we treat PIS as one type of social relationship (self-organization relationship) and seek to understand the relationship between PIS and feedback reactions.

### Perceived Insider Status

Perceived insider status represents the extent to which an employee perceives oneself as an insider within a particular organization (Stamper and Masterson, 2002). In the past, researchers were defining PIS from two viewpoints: an employee’s self-concept and an employee-organization relationship (Dai and Chen, 2015). By using two different perspectives to define PIS, scholars have generally relied on various theories to explain their empirical results.

If PIS was treated as a self-concept, then researchers commonly used the social exchange theory or the inducements and contributions theory to explain their results. Organizations distinguishing between insider and outsider employees may use inducements such as higher leader member exchange (Stamper et al., 2009), empowerment (Chen and Aryee, 2007), benefits, training or promotions to send signals to convince employees that they have achieved insider status. In this branch of studies, PIS was featured as an inducement because those employees who feel a higher level of PIS may think that they are more central and important to the organization. Based on the theory of social exchange, such employees are likely to reciprocate by positively modifying their work behaviors and striving to become more productive or contribute to creative processes in the organization (Sui and Wang, 2013; Hui et al., 2015; Liao, 2015).

When interpreting PIS as a kind of employee-organization relationship, scholars used the theories of organizational socialization and the self-categorization to explain related results (Dai and Chen, 2015). Bauer suggested that organizational socialization could refer to the procedures new employees incorporate to ensure that their knowledge, attitudes and behaviors are accepted by other members of the organization, which indicates a role conversion process of the individual from an outsider to an insider. Individuals seek to form a solid relationship with a group to satisfy their need to belong (Masterson and Stamper, 2003). Once a certain employee-organization relationship has been established, employee behaviors and attitudes will start conforming to group norms. If the relationship is considered unsatisfactory, the employee will not be committed and may even consider leaving the company (Knapp et al., 2014).

Much of previous research explains PIS by using the theory of social exchange. However, few studies have evaluated PIS in terms of outcomes by treating it as a conceptualization related to employee-organization relationships. Following the above arguments, when treating feedback intervention as a form of social interaction, we believe the perception of employee-organization relationship (PIS) may also affect feedback reactions.

### PIS and Feedback Reactions: Perspective of Self-categorization

The theory of self-categorization asserts that the categorization process is fundamental to group formation (Turner et al., 1987). During such a process, group members develop their own levels of inclusiveness that represent different degrees of strength with respect to self-group relationships. Members developing strong self-group relationships are more likely to perceive themselves less as individuals but more as interchangeable exemplars of the group prototypes, which implies that depersonalization of the individual has occurred. When individuals accomplish the construction of strong self-group relationship through the process of depersonalization, they may become more tolerant of dissent within the group (Hornsey et al., 2002). Feedback is a type of message that often conveys dissent regarding a member’s daily behavior. Previous research has found that feedback is typically received in a less defensive manner when the feedback is made by another member of the group rather than by an outsider (Hornsey et al., 2002; Rabinovich and Morton, 2012). This means that the feedback receivers’ perception of group identities (strong and connected self-organization relationship) might influence feedback interpretation. In accordance with the theory of self-categorization, we suggest that different strengths of self-organization relationship will result in different feedback reactions. In this study, PIS represents the strength of self-organization relationship.

According to the self-categorization theory, individuals engage in depersonalization by identifying a group prototype with their self-concept (Turner et al., 1987). Not all group members necessarily enact and encompass all defining features of a group, although certain group members may embody central group characteristics (i.e., central members perceive high insider status), other group members turn out to be more marginal in the sense that they show greater discrepancies between core group attributes and individual characteristics (i.e., marginal members perceive low insider status) (Ellemers and Jetten, 2013). The existence of different levels of depersonalization makes employees feel that they have been accorded a different group status (insider status or outsider status). Achievement of an insider status gives certain group members access to more resources and contributes more to group-derived self-esteem (Gómez et al., 2013). When central members perceiving high insider status receive feedback from their supervisors, they may engage in improved feedback acceptance because they treat feedback as a type of organizational resource and signals standing for future development or promotion.

Certain group members labeled as under-prototypical or marginal may have fewer opportunities to gain resources and respect, so that, they are more likely to perceive themselves as outsiders (Stamper and Masterson, 2002). The value of supervisory feedback among such group members can differ from that for central group members. Marginal members may interpret feedback in a more pessimistic manner; they receive feedback because they did not do well in their position, or they are not accepted by their supervisors. However, as every embedded group member feels the need to belong and hopes to build stronger relationship with the group, certain behaviors will be activated to change a situation when the need arises (Hornsey and Jetten, 2004; Ellemers and Jetten, 2013). Marginalized members might utilize compensatory behaviors by accepting feedback and using feedback for behavior modification. Because feedback conveys other members’ attitudes toward the employee and represents a clear demand to conform to group norms, feedback given to marginal members constitutes useful information that may result in better acceptance by the group.

To summarize, we posit that both marginal members and central members will accept feedback. Central members perceiving high insider status use feedback because it is a resource for future achievement. Marginal members perceiving low insider status accept feedback as a type of compensatory behavior to develop better group inclusion. Based on above statements, we believe that both employees perceiving high PIS (Central members) and low PIS (Marginal members) will have good feedback reactions. In particular, we propose that a U-shape relationship may occur between PIS and feedback reactions. Specifically, when compared to employees that perceive a middle-level of insider status, both employees that perceive a high insider status and employees that perceive a low insider status will have positive feedback reactions.

As noted above, many previous studies and theories have indicated that cognitive feedback reaction (feedback utility) and affective reaction (feedback satisfaction) should be discussed separately. Individuals experiencing low levels of group inclusion may experience more negative emotions because of the lack of feeling that they belong to the organization (Knapp et al., 2014). Hence we postulate that affective feedback reaction is linearly related to PIS. With regard to cognitive feedback reaction, marginal members may become unhappy about feedback and their present state, but they will engage in compensatory behavior (utilize feedback to gain group acceptance) to change it (Gómez et al., 2011; Ellemers and Jetten, 2013). Hence a curvilinear relationship may exist between PIS and cognitive feedback reaction. In sum, we propose the following two hypotheses regarding the correlations between PIS and feedback reactions.

Hypothesis 1a: A U-shape relationship exists between PIS and feedback utility (The Curvilinear Hypothesis).
Hypothesis 1b: A positive relationship exists between PIS and feedback satisfaction (The Linear Hypothesis).

### Mediating Mechanism of Feedback Motivation Attribution

Much research analyzing feedback has indicated that attribution knowledge significantly impacts feedback reactions (Tolli and Schmidt, 2008). In addition to the attribution of feedback information, another type of attribution is not directed to feedback information but toward supervisors. Feedback recipients make assumptions regarding the motivation of the supervisor. Scholars have identified two directions of feedback motivation attribution that are similar to the attribution theory: external and internal (Hempel, 2008). The need to improve job performance or help the company’s competitive position are examples of external attributions related to supervisors’ feedback motivation. Supervisory feedback attributed to these motivations may be perceived as a desire to improve performance and lead to positive outcomes. Feedback attributed to internal supervisory motivations, such as (dis)like or moodiness, may not be perceived as concern with a task or job performance, but might be interpreted as a veiled message concerning declining working relationships. Feedback messages attributed to internal motivations may be viewed in a negative manner and may lead to a decline in motivational and affective levels to work and perform (Leung et al., 2001; Hempel, 2008).

While analyzing the concept of feedback motivation attribution, scholars generally realize that motivation attribution is a concept that stems from social interaction associated with feedback. Previous studies have determined that interpersonal relationships could positively predict affective feedback reaction through mediation of feedback motivation attribution (Leung et al., 2001; Hempel, 2008). The perception of insider status represents the quality of employee-organization relationship. Since it is also a relationship dimension that could be drawn from the group dynamic perspective, it is very likely that feedback motivation attribution may be a possible psychological process linking PIS and feedback reactions.

**FIGURE 1:**
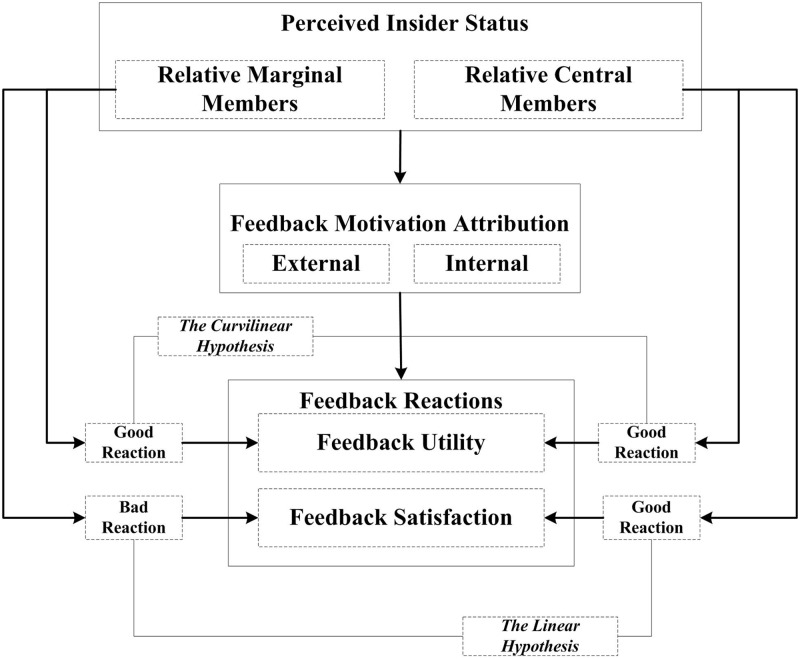
**Research model**.

According to self-categorization theory, when a feedback receiver possesses the group identity and perceive strong self-organization relationship, we may expect that other group members (e.g., supervisor) would be treated as less of a threat to self-identity; they generally possess legitimate identity to convey criticism, and their feedback may be viewed as constructive (Hornsey et al., 2004; Rabinovich et al., 2014). We postulate that either perception of negativity or constructive nature of feedback is a consequence of attribution on feedback deliverer motivation. Therefore, we consider the mediating mechanism as the psychological process that connects PIS and feedback reactions. We now propose the following hypotheses (all hypothesized relations are summarized in **Figure 1**).

Hypothesis 2a: Internal feedback motivation attribution about supervisor mediates the positive relationship of PIS and Feedback Satisfaction.
Hypothesis 2b: Internal feedback motivation attribution about supervisor mediates the positive relationship of PIS and Feedback Utility.
Hypothesis 2c: External feedback motivation attribution about supervisor mediates the positive relationship of PIS and Feedback Satisfaction.
Hypothesis 2d: External feedback motivation attribution about supervisor mediates the positive relationship of PIS and Feedback Utility.

## Materials and Methods

### Sample and Data Collection

To avoid common method bias we utilized the two waves data collecting method to control bias that results from self-reported data. The interval between the two time points is 1 month. The survey was initially conducted September, 2015. The independent variable and mediating variables were collected during the first time point, the dependent variables were collected the following month. The sample of our study included employees of a machinery manufacturing company in the Guangdong province of China.

This research has been performed in accordance with the recommendations of the Science & Technology Research Office of Huazhong University of Science and Technology. There were no unethical behaviors in the research process, and we were exempt from further ethics board approval since the study did not involve human clinical trials or animal experiments. We first got in touch with the human resource director of the firm and then asked whether this survey can be conducted in the company. Upon approval of the human resource manager of the company, 210 questionnaires were distributed, and all were received upon the first wave of data collection. Only those who were willing to participate were recruited. One month later, the same 210 employees were sought out to complete the entire survey and 200 responded. The response rate was 95%. In accordance with basic principles of a valid questionnaire, eight questionnaires were eliminated because they showed similar answering patterns or questionnaires were answered in a paradoxical manner (Winters et al., 1993). The average age of the valid samples was 39.2 years; 48.5% of respondents had an undergraduate education; the average length of tenure was 8.9 years; and 74% of respondents were male.

### Measures

Chinese versions of questionnaires were back-translated into English to verify content and meaning. Because all constructs were used in Chinese context in related studies, we believe the application of certain constructs to Chinese culture background were effective. All constructs were measured using the 7-point Likert-type scale (1 = strongly disagree to 7 = strongly agree).

#### Feedback Utility

The feedback sources were constrained to the feedback comes from the supervisors at the beginning of the questionnaires. Feedback utility was selected as the study object of cognitive feedback reaction. The four-item scale was also used in a study conducted by Jawahar (2010). A sample item was, “I learned a lot from my performance evaluation discussion.”

#### Feedback Satisfaction

The feedback sources were constrained to the feedback comes from the supervisors at the beginning of the questionnaires. Affective feedback reaction was measured by a commonly used method: six-item feedback satisfaction measurement. The scale was adapted from Dobbins’s study (Dobbins et al., 1990). A sample item was, “I am satisfied with my most recent appraisal.”

#### Perceived Insider Status

The six-item scale proposed by Stamper was utilized to measure employee PIS (Stamper and Masterson, 2002). A sample item was, “I feel very much a part of my work organization.”

#### Feedback Motivation Attribution

Two types of feedback motivation attributions about supervisory feedback were measured: external and internal feedback motivation attribution (Hempel, 2008). Both feedback motivation attribution scales contain three measured items. All employees were asked why their supervisor gave them specific feedback. Different motivation attributions were applied and corresponded to the rating agreement. External feedback motivation attribution was measured using items that measure the purpose of the feedback and included: “They want to help improve company productivity,” “Company faces pressure from competitors,” and “They need to pass on information about my performance from other sources (i.e., clients or customers).” Internal feedback motivation attribution was measured using the following items: the supervisor gave feedback “Due to his/her emotions,” “To demonstrate his/her authority,” and “Because he/she dislikes me.”

Control variables were utilized because prior studies have demonstrated that different demographical properties may influence feedback reactions. Demographic variables were selected as the control variables and include gender, age, tenure, and educational level (Geddes and Konrad, 2003). During our data analysis, gender and educational level were coded as dummy variables.

## Result

### Preliminary Analyses

A confirmatory factor analysis was conducted using Amos 7.0 to check the discriminant validity of different scales (Anderson and Gerbing, 1988). The results displayed in **Table 1** indicate that the five-factor model had a better model fit than other construct combination models (χ^2^/df = 2.341; CFI = 0.903, IFI = 0.905, TLI = 0.877; RMSEA = 0.084).

**Table 1:** Confirmatory factor analysis results.

	χ^2^/df	IFI	CFI	TLI	RMSEA
**Five factors model**	2.341	0.905	0.903	0.877	0.084
(PIS, external FMA, internal FMA, feedback utility, and feedback satisfaction)					
**Four factors model**	4.374	0.739	0.734	0.676	0.133
(PIS, external FMA, internal FMA, and feedback reactions)					
**Three factors model**	4.567	0.720	0.716	0.658	0.136
(PIS, feedback motivation attribution, and feedback reactions)					
**Two factors model**	5.574	0.638	0.633	0.561	0.154
(PIS, feedback motivation, attribution and feedback reactions)					
**Single factor model**	6.773	0.542	0.535	0.446	0.173


**Table 2** presents the means, standard deviations, and zero-order Pearson correlations for all key variables and Cronbach’s α for each variable. The results indicated that Cronbach’s α of all the scales used in our study was higher than 0.7 (PIS, 0.875; internal feedback motivation attribution, 0.741; external feedback motivation attribution, 0.869; feedback satisfaction, 0.936; and feedback utility, 0.924), which indicates that the scales have good internal consistency.

**Table 2:** Means, standard deviations, correlations, and reliabilities.

	*M*	*SD*	1	2	3	4	5	6	7	8
(1) PIS	5.95	0.79	(0.87)							
(2) External FMA	5.03	0.98	0.25^∗∗^	(0.74)						
(3) Internal FMA.	2.60	1.15	-0.51^∗∗^	-0.08	(0.86)					
(4) Feedback utility	5.03	1.11	0.41^∗∗^	0.29^∗∗^	-0.28^∗∗^	(0.93)				
(5) Feedback satisfaction	4.64	1.17	0.49^∗∗^	0.18^∗^	-0.36^∗∗^	0.61^∗∗^	(0.92)			
(6) Gender	0.75	0.43	-0.01	0.07	-0.00	0.09	-0.04			
(7) Age	37.12	7.20	-0.01	0.09	0.07	0.00	-0.01	0.06		
(8) Tenure	9.76	5.30	0.05	0.17^∗^	0.03	0.01	0.01	-0.08	0.57^∗∗^	
(9) Education	0.91	0.52	-0.12	-0.24^∗∗^	-0.01	-0.11	-0.12	0.12	-0.11	-0.25^∗∗^


**N* = 192; ^∗^*p* < 0.05, ^∗∗^*p* < 0.01.*

### Tests of Hypotheses

Hierarchical regression analysis was applied to test the hypotheses. The results of hierarchical regression are presented in **Table 3**. The results as model 3, 6 demonstrated that PIS positively predicted feedback utility (β = 0.41, *p* < 0.01), feedback satisfaction (β = 0.49, *p* < 0.01). After centering the independent variable (PIS), we analyzed the effect of PIS square on feedback reactions. Results as model 4, 7 showed that PIS square was positively related to feedback utility (β = 0.18, *p* < 0.01), which suggests a U-shape relationship between PIS and feedback utility, this result disappeared on feedback satisfaction (β = 0.03, n.s.). Hence, we can conclude that Hypothesis 1a and Hypothesis 1b were supported.

**Table 3:** Results of regression analysis for the effect of PIS on feedback motivation attribution (FMA) and feedback reactions.

Variables	External FMA	Internal FMA	Feedback utility			Feedback satisfaction		
				
	Model 1	Model 2	Model 3	Model 4	Model 5	Model 6	Model 7	Model 8
Gender	0.11	-0.00	0.11	0.10	0.08	-0.03	-0.03	-0.04


Age	0.00	0.05	0.01	-0.00	0.01	0.01	0.01	0.02


Tenure	0.12	0.02	-0.03	0.00	-0.05	-0.05	-0.05	-0.06


Education	-0.20^∗∗^	-0.05	-0.08	-0.08	-0.04	-0.07	-0.07	-0.07


**Independent Variables**								
PIS	0.23^∗∗^	-0.52^∗∗^	0.41^∗∗^	0.53^∗∗^	0.31^∗∗^	0.49^∗∗^	0.50^∗∗^	0.40^∗∗^
PIS^2^				0.18^∗^			0.03	
**Mediators**								
External FMA					0.21^∗∗^			0.07
Internal FMA					-0.10			-0.16^∗^
***R*^2^**	0.14^∗∗^	0.27^∗∗^	0.18^∗∗^	0.21^∗∗^	0.23	0.26^∗∗^	0.26^∗∗^	0.28^∗∗^
**Δ*R*^2^**	0.14^∗∗^	0.27^∗∗^	0.16^∗∗^	0.02^∗∗^	0.21^∗∗^	0.26^∗∗^	0.00	0.26^∗∗^


*^∗^*p* < 0.05, ^∗∗^*p* < 0.01; Standardized regression coefficients are reported.*

We tested the mediating mechanism of feedback motivation attribution through applying Baron and Kenny’s multi-step regression procedure (Baron and Kenny, 1986). According to Baron and Kenny (1986), “full” mediation occurs when meeting three demands. First, the independent variable must affect the mediator variable. Second, the independent variable must be demonstrated to affect the dependent variables. Third, the mediator variable must affect the dependent variables. Lastly and most importantly, the independent variable must no longer be significant when the mediator variable is included in the regression equation. “Partial” mediation occurs similar to “full” mediation, except that “partial” mediation does not require the effect of the independent variable to be insignificant. Following these three procedures, the analysis demonstrated that PIS positively predicted external feedback motivation attribution (β = 0.23, *p* < 0.01) and negatively predicted internal feedback motivation attribution (β = -0.52, *p* < 0.01). Results of model 5 in **Table 3** showed that, external feedback motivation attribution was positively related to feedback utility (β = 0.21, *p* < 0.01), while internal feedback motivation attribution was not significantly related to feedback utility (β = -0.10, n.s.). As the effect of PIS on feedback utility decreased (β = 0.31, *p* < 0.01), we can conclude that external feedback motivation attribution partially mediated relationship between PIS and feedback utility. But the mediating effect of internal feedback motivation was not significant. Results of model 8 showed that internal feedback motivation attribution was negatively related to feedback satisfaction (β = -0.16, *p* < 0.01), while external feedback motivation attribution was not significantly related to feedback satisfaction (β = -0.07, n.s.). As the effect of PIS on feedback satisfaction also declined (β = 0.4, *p* < 0.01), we can conclude that internal feedback motivation attribution was the valid partial mediator but not external feedback motivation attribution.

In addition, to test the indirect effect, we used bias corrected bootstrapping techniques (1000 replications). The results of **Table 4** showed that PIS had an indirect effect on feedback utility via external feedback motivation attribution (indirect effect = 0.07, 95% CI = [0.02, 0.16] excludes zero). However, this results could not be generalized to internal feedback motivation attribution (indirect effect = 0.07, 95% CI = [-0.04, 0.24] includes zero). For feedback satisfaction, we found the reversed patterns of results, PIS had an indirect effect on feedback satisfaction via internal feedback motivation attribution (indirect effect = 0.13, 95% CI = [0.01, 0.28] excludes zero), however, this results could not be generalized to external feedback motivation attribution (indirect effect = 0.02, 95% CI = [-0.02, 0.10] includes zero). Hence, hypothesis 2a, 2d were supported, but hypothesis 2b, 2c were not supported.

**Table 4:** Mediation effect results.

Model	Mediation Effect	95% confidence interval
PIS → external feedback motivation attribution → feedback utility	0.07	[0.02, 0.16]
PIS → internal feedback motivation attribution → feedback utility	0.07	[-0.04, 0.24]
PIS → external feedback motivation attribution → feedback satisfaction	0.02	[-0.02, 0.10]
PIS → internal feedback motivation attribution → feedback satisfaction	0.13	[0.01, 0.28]


### Supplemental Hypothesis

**FIGURE 2:**
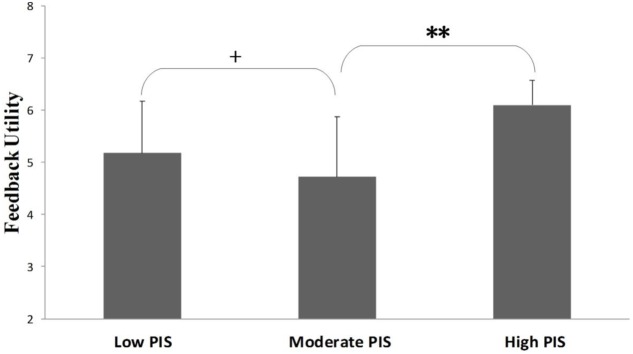
**Feedback utility means on different PIS groups.** ^∗∗^*p* < 0.01, ^+^*p* < 0.1.

Supplemental analysis was conducted to confirm that there appears the curvilinear effect between PIS and feedback utility. According to previous study we generated predicted values at the mean and plus and minus one standard deviation on the PIS score to represent the moderate PIS, high PIS, and low PIS groups (Suls et al., 2002). After the group classification, we used one-way ANOVA to compare the feedback utility means of different groups. Results of multiple comparison in one-way ANOVA showed that when taking feedback utility as the dependent variable, means of feedback utility from low PIS group was higher than moderate PIS group (*M*_low_ = 5.18, *SD*_low_ = 1.00; *M*_moderate_ = 4.72, *SD*_moderate_ = 1.15. *p* < 0.1). Meanwhile means of feedback utility from moderate PIS group was lower high PIS group (*M*_moderate_ = 4.72, *SD*_moderate_ = 1.15; *M*_high_ = 6.10, *SD*_high_ = 0.47. *p* < 0.01), the pattern of the means difference additionally implicate that there is the U-shape effect between PIS and feedback utility. The multiple comparison result suggested that mean difference between low PIS group and moderate PIS group only reached marginal statistical significance, in order to provide more evidence, we apply the *t*-test to inspect the difference between low PIS group and moderate PIS group. The *t*-test result further suggested the feedback utility mean of low PIS group was higher than moderate PIS group (*t* = 2.023, *p* < 0.05) (**Figure 2**).

## Discussion

Our analysis of empirical data led to three sets of meaningful results. First, a positive association was indicated between PIS and feedback reactions. In addition to the positive relationships, it was demonstrated that a U-shaped relationship exists between PIS and feedback utility. Second, the analysis indicated that the relationship between PIS and feedback reactions was not a direct effect. Feedback motivation attribution was one of the psychological mechanisms mediating the relationship. Third, we found that different correlation patterns exist between affective feedback reaction and cognitive feedback reaction. Specifically, the speculation of a U-shaped relationship between PIS and feedback utility was confirmed. However, no such relationship was found between PIS and affective feedback reaction. The mediating mechanisms of affective reaction and cognitive reaction were also different in nature. The effect on affective reactions was partially mediated by internal motivation attribution while the effect on cognitive reaction was partially mediated by external motivation attribution.

### Theoretical Implications

The results of this study have several theoretical contributions pertaining to feedback reactions. First, this study has applied the self-categorization theory as the overarching theory (Turner et al., 1987). Several previous studies had pointed out that self-process could be a major predictor of feedback reactions. Feedback intervention theory incorporates self-esteem into their theoretical frameworks to explain how certain types of feedback might direct attention toward self-esteem and interfere with feedback acceptance (Kluger and DeNisi, 1996). Unlike feedback intervention theory which focuses only on trait relevant aspects of self-process, we have utilized the perspective that considers interaction between self-identity and group and found that individuals’ perception about self-organization relationships would also influence feedback reactions. This result points to the need for deeper studies on self-process and feedback. The usage of self-categorization theory not only fills the gap in terms of self-process studies, but offers new understanding by treating feedback intervention as the social interaction process (Hempel, 2008). Specifically, we choose self-organization relationships (PIS) as the independent variable and evaluate how this kind of social relationship can also exert influence on feedback reactions.

Secondly, some scholars have delineated the identities of insider and outsider as research objects (Hornsey and Jetten, 2004; Esposo et al., 2013) and found that the feedback receiver is more likely to reject feedback provided by a complete outsider. This result can be of practical use while solving problems stemming from between-group conflicts, e.g., racial discrimination (Ariyanto et al., 2006). However, in reality, we are dealing mostly with feedback derived from in-group members (supervisory feedback). Thus, in the organizational feedback context, it is more urgent to study individual’s self-categorization process. Our study has highlighted the importance of understanding in-group dynamics as a mechanism of feedback processing. Still, future studies should try to explore the specific mechanism, such as different emotional perceptions. According to previous literature, marginal group members experience negative emotions such as sadness or pain when they are not accorded insider status in their organization (Eisenberger et al., 2003). However, feedback from an outsider group may symbolize a potential threat and stimulate anger rather than pain and sadness (Esposo et al., 2013). Consequently, we suggest that future studies seek to reveal a more detailed emotional mechanism that might be simultaneously triggered by feedback intervention and in-group dynamics.

Finally, we had hoped to make new theoretical contribution by comparing distinct correlation patterns of affective and cognitive feedback reactions. According to the cognitive theory of emotion behavior, emotion and cognition affect behavior in interactional ways (Oatley and Johnson-laird, 1987). After rational cognitive evaluation, while facing supervisory feedback, employees may still accept the beneficial feedback even though they are not happy about it (Johnson and Connelly, 2014). Our study has demonstrated a range of correlation patterns both with respect to the main effect and mediating mechanism. It has been shown that the main effect consists of a curvilinear relationship between PIS and feedback utility along with a linear relationship between PIS and feedback satisfaction. This result is in agreement with findings from several previous studies proposing that marginal members experiencing unsatisfactory emotions might strive harder to change the situation (Ellemers and Jetten, 2013). Surprisingly, we have also found different mediating mechanisms with regard to feedback utility and feedback satisfaction: external attribution about supervisors’ feedback motivation mediates the relationship between PIS and feedback utility while internal attribution mediates the relationship between PIS and feedback satisfaction. This implies that constructive attribution (external attribution) about others’ behavior is likely to lead to rational reactions (feedback utility), while non-constructive attribution about supervisor might lead to rebelling emotions rather than rational cognition. Our finding is in agreement with studies which have found that, when people make non-constructive attribution about another one, they can be trapped by the unsatisfactory emotional experience and won’t bother to rationally accept information that might be useful (Hornsey et al., 2002).

### Practical Implications

Our study has confirmed the relationship between self-organization and feedback reactions, which implies that predetermined social information also plays a role in affecting feedback intervention (Levy and Williams, 2004; Pichler, 2012). Although some scholars have emphasized the social elements of performance appraisal, their studies were limited to the social elements involved in the social interactions between the rater and the ratee (Leung et al., 2001). Therefore, organizations should keep in mind that social-cues might distort interpretation of feedback information. Meanwhile, in order to effectively avoid the interference and guide employees’ attention toward task-related aspects of feedback, organizations should design feedback system conveying the objective and fair information rather than subjective and emotional information.

Through our analysis, we have demonstrated that managers may improve feedback effectiveness by caring more for employees and making them feel included in the group. Managers should not be afraid to provide feedback information to employees feeling that they have been rendered marginal in the group. Feedback to this type of employee symbolizes an opportunity for change and acceptance as an in-group member. However, managers should be cognizant of the possibility that marginal members may be more emotional and vulnerable to feedback information. The ultimate suggestion for general management practice is that organizations should take action to help staff feel that they belong; this could be a vital part of the work group to reduce conflict and work-related stress that can add burdens and extra work for managers.

### Limitations and Future Research

The results of this study should be interpreted with caution. First, since we had conducted this study in China, it is not clear how many of the results can be generalized to the Western context. It is possible that the interference of social information during feedback processing is more apparent in a Chinese organization. China is a relationship-oriented society (Tsui and Farh, 1997) and, because Chinese develop a stronger dependent self-concept, processing social cues is of particular importance there (Lee et al., 2000; Van De Vliert et al., 2004; Nakashima et al., 2008). Many scholars have demonstrated that Chinese managers find it difficult to provide feedback because they are unable to overlook the critical influence of social relationships and other social cues (Hempel, 2008). Thus, we recommend that future researches explore the cultural differences as the boundary condition that might influence the proposed model in this research.

Secondly, all our variables were measured on the basis of information collected through self-reports. Therefore, although a two-time data collection method was utilized to avoid this bias, common method bias is a concern. Future researchers may implement an experimental method to investigate the causal relationship between group inclusion and feedback reactions. Another limitation of this study is that the effects of the feedback sign (positive feedback or negative feedback) were not verified (Vancouver and Tischner, 2004), although usage of feedback reaction scales could measure the basic acceptance of feedback. Since differences in the mechanisms underlying negative feedback and positive feedback have not been revealed by the present study, it may be unwise to extend the deduction to studies regarding negative feedback. It is our hope that future studies can clarify how group inclusion influences positive feedback and negative feedback processing.

Meanwhile, we recommend several directions for promoting research on feedback studies. Since the present study has evaluated satisfactory attitude as the emotional reactions toward feedback, future studies could retest the present model by considering discrete emotions as different affective feedback reaction. Likewise, since updating referents has pointed to discrete emotions, even negative ones (e.g., anger, guilt) could predict various afterward behaviors of feedback intervention (Johnson and Connelly, 2014). In addition, future studies could explore the consequences of feedback reactions. Because feedback reactions constitute a construct of worker’s attitude, the concept cannot directly represent the effect of feedback intervention. Future investigators could profitably focus more on the relationship between feedback reactions and worker’s job outcomes (performance and other work attitudes) while developing more complete models of the effects of feedback intervention.

## Conclusion

Drawing on self-categorization theory, the present paper has discovered employee perceptions regarding their insider status as one of the antecedents of feedback reactions. In the process, it has demonstrated that different psychological mechanisms should be considered while evaluating cognitive feedback reactions and effective feedback reactions. By utilizing the linear and curvilinear relationships identified between PIS and feedback reactions, organizations should be able to help employees to accept performance feedback effectively and thus foster better learning attitudes.

## Ethics Statement

This study is a investigation happened in a company under permission of the manager. The survey started in September of 2015, the sample of our study is contributed from a Machinery manufacturing company in Guangdong province of China.

## Author Contributions

XC designed the study, carried it out, analyzed the results, and wrote the manuscript. JL designed the study and carried it out. WW analyzed the results and wrote the manuscript. WZ wrote the manuscript.

## Conflict of Interest Statement

The authors declare that the research was conducted in the absence of any commercial or financial relationships that could be construed as a potential conflict of interest.
